# Updated clinical guidelines experience major reporting limitations

**DOI:** 10.1186/s13012-017-0651-3

**Published:** 2017-10-12

**Authors:** Robin W.M. Vernooij, Laura Martínez García, Ivan Dario Florez, Laura Hildago Armas, Michiel H.F. Poorthuis, Melissa Brouwers, Pablo Alonso-Coello

**Affiliations:** 1 Iberoamerican Cochrane Centre, Biomedical Research Institute Sant Pau (IIB Sant Pau), Barcelona, Spain; 2 Department of Health Research Methods, Evidence and Impact; McMaster University, Hamilton, Canada; 3 Department of Pediatrics, University of Antioquia, Medellin, Colombia; 4 University Medical Center Utrecht, Utrecht, The Netherlands; 5 Department of Oncology, McMaster University, Hamilton, Canada; 6 CIBER of Epidemiology and Public Health (CIBERESP), Madrid, Spain

**Keywords:** Checklist/standards, Guideline [publication type], Publishing/standards

## Abstract

**Background:**

The Checklist for the Reporting of Updated Guidelines (CheckUp) was recently developed. However, so far, no systematic assessment of the reporting of updated clinical guidelines (CGs) exists. We aimed to examine (1) the completeness of reporting the updating process in CGs and (2) the inter-observer reliability of CheckUp.

**Methods:**

We conducted a systematic assessment of the reporting of the updating process in a sample of updated CGs using CheckUp. We performed a systematic search to identify updated CGs published in 2015, developed by a professional society, reporting a systematic review of the evidence, and containing at least one recommendation. Three reviewers independently assessed the CGs with CheckUp (16 items). We calculated the median score per item, per domain, and overall, converting scores to a 10-point scale. Multiple linear regression analyses were used to identify differences according to country, type of organisation, scope, and health topic of updated CGs. We calculated the intraclass coefficient (ICC) and 95% confidence interval (95% CI) for domains and overall score.

**Results:**

We included in total 60 updated CGs. The median domain score on a 10-point scale for presentation was 5.8 (range 1.7 to 10), for editorial independence 8.3 (range 3.3 to 10), and for methodology 5.7 (range 0 to 10). The median overall score on a 10-point scale was 6.3 (range 3.1 to 10). Presentation and justification items at recommendation level (respectively reported by 27 and 38% of the CGs) and the methods used for the external review and implementing changes in practice were particularly poorly reported (both reported by 38% of the CGs). CGs developed by a European or international institution obtained a statistically significant higher overall score compared to North American or Asian institutions (*p* = 0.014). Finally, the agreement among the reviewers on the overall score was excellent (ICC 0.88, 95% CI 0.75 to 0.95).

**Conclusions:**

The reporting of updated CGs varies considerably with significant room for improvement. We recommend using CheckUp to assess the updating process in updated CGs and as a blueprint to inform methods and reporting strategies in updating.

**Electronic supplementary material:**

The online version of this article (10.1186/s13012-017-0651-3) contains supplementary material, which is available to authorized users.

## Background

Clinical guidelines (CGs) are defined as ‘statements that include recommendations intended to optimise patient care, that are informed by systematic reviews of evidence and an assessment of the benefits and harms of alternative care options’ [1]. Scientific knowledge is in constant evolution [2, 3]; therefore, surveillance of the new evidence is required to ensure the trustworthiness of clinical guidelines (CGs) [4–8].

Updating CGs is an iterative process with a systematic and explicit methodology that involves identifying and reviewing new evidence not included in the original version of a CG [9]. The fundamental stages of the updating process are (1) prioritising of CGs and clinical questions [10, 11], (2) identifying of new evidence [8, 12, 13], (3) assessing the impact of the new evidence and decision to update [4, 8], (4) reviewing and—if necessary—modifying the recommendations [14–16], and (5) reporting updated recommendations [17]. Currently, there is no consensus about the optimal methodology to maintain CGs up-to-date [11, 18, 19].

The reporting of updated CGs is a process within an updating strategy that communicates users about the methods and changes in an updated CG [9]. So far, there is limited guidance on the reporting of the updating process [19]. To address this gap, we recently developed the Checklist for the Reporting of Updated Guidelines (CheckUp) [20]. The aim of CheckUp is to evaluate the completeness of reporting in updated CGs [20]. CheckUp can be used (1) to inform about strategies for updating CGs and their reporting requirements (CG developers), (2) to assess the reporting of updated CGs (interested CG users), and (3) to complete as a publication requirement of updated CGs (editors of scientific journals that publish CGs) [20]. Although CheckUp has been already included in some methodological handbooks and methodological studies [21, 22], it has not been yet formally implemented.

To our knowledge, updated CGs have not been systematically reviewed to assess the completeness of reporting the updating process. An overview of the current status could be informative for the CG community. Therefore, the objectives of our study were (1) to assess the completeness of reporting the updating process of updated CGs using CheckUp and (2) to explore the inter-observer reliability of CheckUp.

## Methods

### Study design

We conducted a systematic assessment of the reporting of the updating process in a sample of updated CGs using CheckUp. We followed the Preferred Reporting Items for Systematic Reviews and Meta-Analyses (PRISMA) guideline to the extent it was applicable to our study [23].

### Information sources and search strategy

We searched in MEDLINE (accessed through PubMed), the G-I-N library (http://www.g-i-n.net), and the National Guidelines Clearinghouse (NGC) (https://www.guidelines.gov) in August 2016 for updated CGs published during 2015. The search strategy can be found in Additional file 1.

### Inclusion criteria

We included all updated CGs published in 2015 (as the most recent year prior to publication of CheckUp) which met the following criteria: (1) developed by a professional society, (2) search strategy using at least one bibliographic database, (3) reporting at least one recommendation, (4) updated version of a previous version of the same CG (including a reference to a previous version of the CG), and (5) published in English.

### Study selection

Two reviewers (RV, IDF, LHA, or MHFP) independently screened the titles and abstracts to identify potentially eligible references. We obtained the full-text articles of the potentially eligible references for further assessment. Disagreements were solved by consensus and, if necessary, with the help of a third reviewer (LMG).

### Data extraction

CheckUp is a checklist consisting of 16 items that examine the reporting of the updating process in updated CGs [20]. CheckUp consists of three domains: (1) presentation of the updated CG (6 items), (2) editorial independence (3 items), and (3) the methodology of the updating process (7 items).

Three reviewers (RV, IDF, LHA, or MHFP) independently evaluated each CG with CheckUp, and whenever the included CGs referred to supplemental documents (e.g. methodological manuals or appendices), these documents were reviewed for additional information.

Furthermore, we collected the following information regarding: (1) the institution that updated the CG (name, country, and type of organisation), (2) the scope of the updated CG (diagnosis, management, prevention, screening, or treatment), and (3) the health topic of the updated CG.

### Data analysis

We calculated summary statistics to provide quantitative information about the institution that updated CGs and CheckUp scores. We calculated item scores (absolute frequencies and proportions) by summing up the updated CGs that reported each item. We calculated domain scores (median and range) by summing up all scores of the individual items for each domain: presentation of the updated CG (6 items), editorial independence (3 items), and the methodology of the updating process (7 items). Additionally, we calculated the overall score (median and range) by summing up all scores of the individual items. Both domain scores and total scores were converted to a 10-point scale.

To identify potential predictors, we used multiple linear regression to test whether the overall score (dependent variable) differed between CG institution’s country, type of organisation, objective of the CG, and CG topic (independent variables).

We calculated the intraclass coefficient (ICC) with its 95% confidence interval (CI) as an indicator of the overall agreement between the three reviewers for each item. According to the scale proposed by Landis and Koch, the degree of agreement between 0.00 and 0.20 was considered poor, from 0.21 to 0.40 fair, from 0.41 to 0.60 moderate, from 0.61 to 0.80 substantial, and from 0.81 to 1.00 almost perfect [24].

We accepted *p* values of less than 0.05 as significant. We performed the analyses using SPSS version 22.0 (SPSS Inc., Chicago, IL, USA).

## Results

### Selection of updated clinical guidelines

**Fig. 1:**
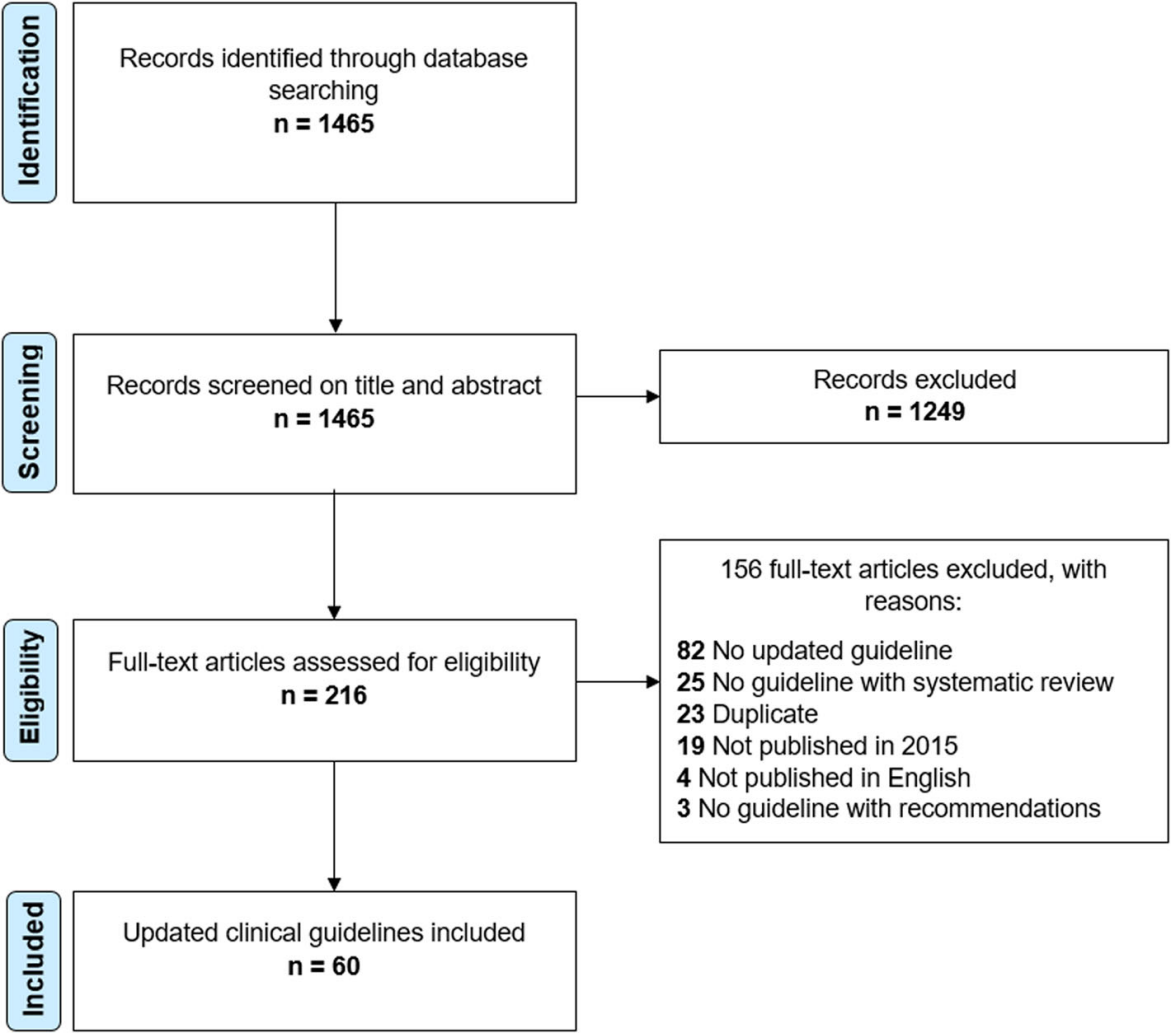
Flow diagram of the screening process

The screening process is summarised in a flow diagram (Fig. 1). We initially identified 1465 references and excluded 1249 references after examining their titles and abstracts. We reviewed 216 full-text articles and excluded 156 references (Additional file 2). Finally, we included 60 updated CGs [25–84].

### Characteristics of included clinical guidelines

Most institutions responsible for updating the CGs were North American (61.7%; 37/60) and scientific/professional societies (46.7%; 28/60) or public institutions (43.3%; 26/60) (Table 1). In total, 25 (41.7%; 25/60) of the included CGs addressed the management of a specific disease. Other CGs address solely the treatment (25.0%; 15/60), screening (15.0%; 9/60), diagnosis (11.7%; 7/60), or prevention (6.7%; 4/60) of a healthcare problem. The clinical area of the included CGs varied widely, with oncology (26.7%; 16/60) the most common.

**Table 1:** Characteristics of the updated clinical guidelines

	*n* (%)
Institution	
Country	
− North America	37 (61.7)
− Europe	17 (28.3)
− Asia	4 (6.7)
− International	2 (3.3)
Type of organisation	
− Scientific/professional society	28 (46.7)
− Public institution	26 (43.3)
− Other (Federal institute, NGO)	6 (10.0)
Updated clinical guidelines	
Scope	
− Management	25 (41.7)
− Treatment	15 (25.0)
− Screening	9 (15.0)
− Diagnosis	7 (11.7)
− Prevention	4 (6.7)
Health topic	
− Oncology	16 (26.7)
− Public health	5 (8.3)
− Internal medicine	3 (5.0)
− Mental health	3 (5.0)
− Others	33 (55.0)

### Domain scores

#### Presentation of the updated CG

**Fig. 2:**
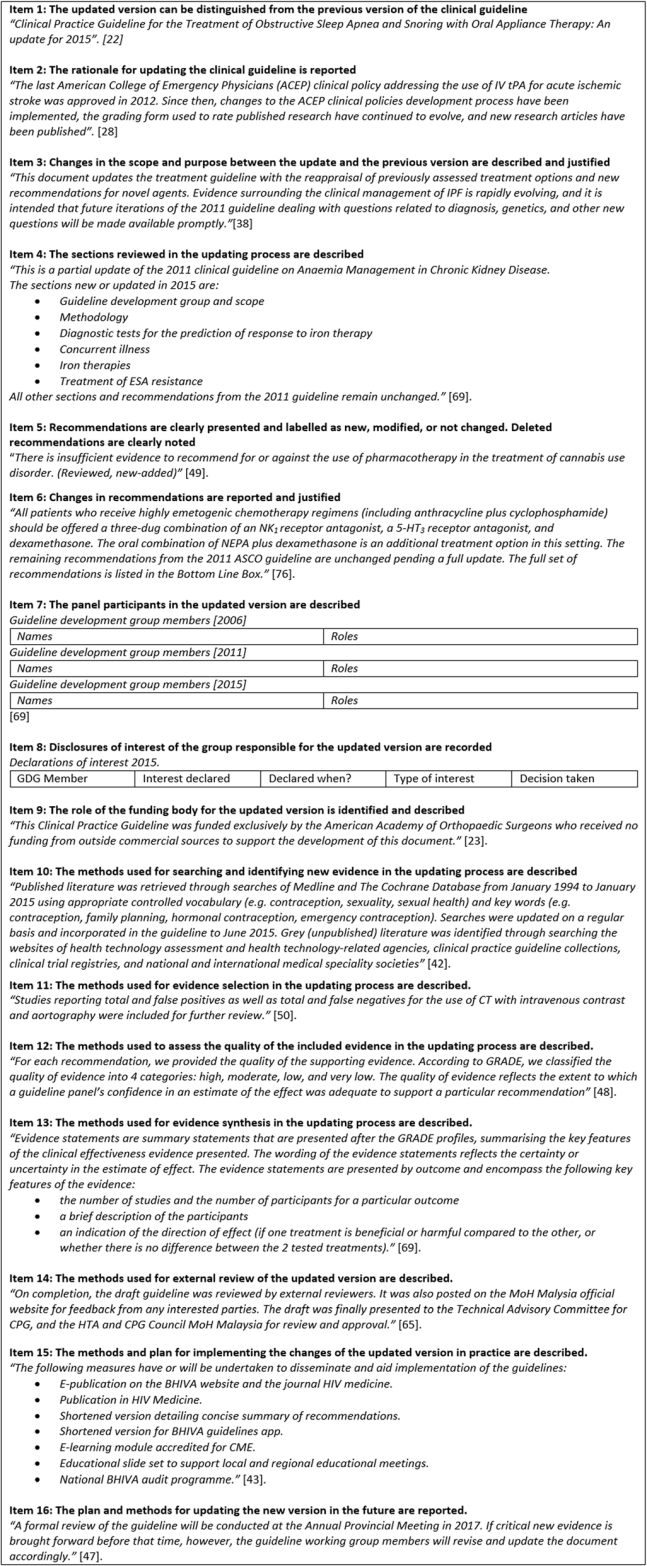
Reporting examples of the included updated CGs

All of the included updated CGs could be distinguished from their predecessors since this was one of the eligibility criteria. The included CGs often used the term ‘update’, ‘version’, or the year of publication (i.e. 2015) in their title (Table 2, Fig. 2).

**Table 2:** Item scores

	Updated CGs reporting each item *n* (%)
Presentation of the updated clinical guideline	
Item 1: The updated version can be distinguished from the previous version of the clinical guideline.	60 (100)
Item 2: The rationale for updating the clinical guideline is reported.	37 (61.7)
Item 3: Changes in the scope and purpose between the update and the previous version are described and justified.	34 (56.7)
Item 4: The sections reviewed in the updating process are described.	40 (66.7)
Item 5: Recommendations are clearly presented and labelled as new, modified, or not changed. Deleted recommendations are clearly noted.	16 (26.7)
Item 6: Changes in recommendations are reported and justified.	23 (38.3)
Editorial independence	
Item 7: The panel participants in the updated version are described.	57 (95.0)
Item 8: Disclosures of interest of the group responsible for the updated version are recorded.	58 (96.7)
Item 9: The role of the funding body for the updated version is identified and described.	30 (50.0)
Methodology of the updating process	
Item 10: The methods used for searching and identifying new evidence in the updating process are described.	49 (81.7)
Item 11: The methods used for evidence selection in the updating process are described.	47 (78.3)
Item 12: The methods used to assess the quality of the included evidence in the updating process are described.	46 (76.7)
Item 13: The methods used for evidence synthesis in the updating process are described.	28 (46.7)
Item 14: The methods used for external review of the updated version are described.	23 (38.3)
Item 15: The methods and plan for implementing the changes of the updated version in practice are described.	23 (38.3)
Item 16: The plan and methods for updating the new version in the future are reported.	24 (40.0)

One guideline is rated as not applicable

More than half of the updated CGs included the rationale for updating (61.7%; 37/60), described changes in the scope and purpose between the updated CG and its predecessor (56.7%; 34/60), and reported the reviewed sections (66.7%; 40/60) (Table 2, Fig. 2).

At the recommendation level, 26.7% (16/60) of the included CGs clearly labelled the recommendations as new, modified, or not changed, and 38.3% (23/60) justified the changes. The justifications for changes commonly included a description of the new evidence that triggered the change in the recommendation and the changes between the new and old version of the recommendations (Table 2, Fig. 2).

The median score of the presentation domain on a 10-point scale was 5.8 (range 1.7 to 10), and the agreement among the three reviewers was adequate (ICC 0.854; 95% CI 0.701 to 0.941) (Table 3).

**Table 3:** Domains, overall, and agreement scores

	Score^a^ median (range)	Agreement ICC (95% CI)
Domain		
− Presentation of the updated CG	5.8 (1.7–10)	0.854 (0.701–0.941)
− Editorial independence	8.3 (3.3–10)	0.724 (0.534–0.860)
− Methodology of the updating process	5.7 (0–10)	0.886 (0.771–0.952)
Overall	6.3 (3.1–10)	0.880 (0.749–0.952)

^a^10-point scale (10 as the best possible score)

*ICC* intraclass coefficient, *CI* confidence interval

#### Editorial independence

Almost all included CGs described the panel participants in the updated version (95.0%; 57/60) and their respective conflicts of interest (96.7%; 58/60) (Table 2, Fig. 2). However, half of the updated CGs did not report the entity and/or the role of the funding body that financed the updated version (50.0%; 30/60).

The median score of the editorial independence domain on a 10-point scale was 8.3 (range 3.3 to 10), and the agreement among the three reviewers was adequate (ICC 0.724; 95% CI 0.534 to 0.860) (Table 3).

#### Methodology of the updating process

Most of the included CGs reported the methods used for searching and identifying new evidence (81.7%; 49/60), selecting the evidence (78.3%; 47/60), and assessing the quality of the included evidence (76.7%; 46/60) (Table 2, Fig. 2). However, the methods for synthesising the evidence (46.7%; 28/60), external review (38.3%; 23/60), implementing the changes of the updated version in practice (38.3%; 23/60), or updating the new version (40.0%; 24/60) were reported less often in the included CGs.

The median score of the methodology domain on a 10-point scale was 5.7 (range 0 to 10), and the agreement among the three reviewers was adequate (ICC 0.886; 95% CI 0.771 to 0.952) (Table 3).

### Overall score

The median overall score on a 10-point scale was 6.3 (range 3.1 to 10), and the agreement among the three reviewers was adequate (ICC 0.880; 95% CI 0.749 to 0.952) (Table 3).

CGs developed by a European or International institution obtained a higher overall score compared to North American or Asian institutions (*p* = 0.014) (Table 4). No significant differences in the overall score were found between CG differing in the type of organisation, scope, or topic.

**Table 4:** Overall scores stratified by characteristics of the updated clinical guidelines

	Overall score^a^ median (range)	*p value*
Institution		
Country		
− Europe	8.1 (4.4–10.0)	0.014
− International	7.8 (6.9–8.8)	
− Asia	5.6 (3.8–6.3)	
− North America	5.6 (3.1–8.1)	
Type of organisation		
− Public institution	6.3 (3.1–10.0)	0.617
− Scientific/professional society	6.3 (3.1–8.8)	
− Other (Federal institute, NGO)	4.4 (3.8–8.1)	
Updated clinical guidelines		
Scope		
− Diagnosis	8.1 (5.0–9.4)	0.097
− Prevention	5.6 (4.4–6.3)	
− Management	6.3 (3.1–10.0)	
− Treatment	6.3 (4.4–8.8)	
− Screening	3.8 (3.1–8.1)	
Health topic		
− Mental health	6.9 (5.0–8.1)	0.099
− Oncology	6.3 (3.8–9.4)	
− Internal medicine	6.3 (5.6–8.1)	
− Public health	3.8 (3.1–3.8)	
− Others	6.3 (3.1–10.0)	

^a^10-point scale (10 as the best possible score)

## Discussion

### Main findings

Our study is the first systematic assessment of the reporting of the updating process in updated CGs using CheckUp. The presentation and methodology domains were reported less completely than the editorial independence domain. Particularly, the items regarding the presentation and justification of the updating process at recommendation level and the methods used for evidence synthesis, external review, implementing, and future updating were poorly reported. Both the domains and overall scores of the included CGs were highly variable. We identified only two (3.3%) CGs with a perfect score (10-point overall score 10) [60, 74].

We observed an adequate ICC reliability between the three reviewers. The lowest ICC was found for the editorial independence domain, but the ICC domain score was still considered adequate. This was mainly due to some CGs that reported the panel participants and their conflicts of interest for those that were responsible for updating the CG; however, they failed to report the same information for those who were responsible for developing the preceding CG.

### Our results in the context of previous research

#### Presentation of updated CGs

Previous research showed that there was no clear improvement in the reporting or methodological quality after updating systematic reviews [85]. Similarly, Hasenfield et al. found that updated CGs were of worse methodological quality compared to their previous version [86]. Few studies have evaluated the optimal presentation formats of CGs in general [87, 88]. Similarly, regarding the updating process of CGs, a wide variability in the formats used to present updated recommendations has been reported by our group [17]. In the field of systematic reviews, Newberry et al. [89] evaluated different formats for presenting the results of updated systematic reviews. One of their conclusions was that different interest groups have different information needs. For example, health managers preferred to have access to all data and the analysis of a systematic review (the original and the updated), whereas clinicians prefer a synthesis that clearly shows what has been changed [89].

In our study, we have identified that, in particular, the presentation of updated recommendations is not optimal, with recommendations often not presented or not clearly labelled as new, modified, or not changed. This might confuse readers who might not be able to identify which recommendations are updated and which ones remain identical. Additionally, the modifications conducted in recommendations are often not described nor justified.

#### Reporting the editorial independence

The same principle regarding editorial independence for developing new CGs should be applied to the updating process [90]. Previous studies, in which the quality of CGs was reviewed with the Appraisal of Guidelines for Research and Evaluation II (AGREE II) instrument, have observed low scores in the domain of editorial independence [91, 92]. We found similar results for the source of funding. However, most of the included updated CGs in our study reported the panel members and their conflicts of interest.

#### Reporting the methodology of the updating process

Until now, most of the methodological research regarding the updating process of CGs concerned the identification and assessment of new evidence (described commonly as the surveillance process) [18]. However, the complete updating process, including the presentation and justification of the updating process at recommendation level, has received less attention. CG developers possibly assume that the complete updating process is equal to the development process of the initial CG [19]. This could explain why the items that have a certain overlap with the development process (i.e. search strategy, evidence selection, and quality assessment) have higher scores compared to the updating items that are methodologically different from the development process (i.e. synthesis, external review, implementation of changes, and updating in the future) of the initial CG. Although the methods for developing CGs evolve rapidly [93], the updating process still does not follow this progress correspondingly [18, 19, 94, 95].

When updating CGs, developers need to pay special attention to the implementation implications of the changes introduced in updated CGs [96]. This can be done by exploring facilitators and barriers, by developing supporting materials, or by providing audit criteria [97]. Recently, GRADE has published Evidence to Decision frameworks to support developers to systematically consider this aspect and other criteria [98]. As living CGs become more common practice [99], developers will need to assess to what extent more frequent changes in recommendations impact their implementability and optimisation of patient care.

### Strengths and limitations

Our study has several strengths. We followed a rigorous and transparent approach and developed a protocol that is available from the authors on request. Additionally, three reviewers independently conducted the assessment of the included CGs and adequate agreement was found.

Our study has some limitations. It is possible that we did not identify all updated CGs that would meet our inclusion criteria due to suboptimal indexing of CGs in biomedical databases, which may limit the representativeness of the results. Additionally, one eligibility criterion was also an item from the checklist, which might have led to the inclusion of more high-quality updated CGs. Consequently, our results might be an overestimate, and the actual reporting be actually worse than our findings.

### Implications for practice and research

When CG developers are interested in updating CGs, we suggest firstly assessing the quality of CGs using the AGREE II instrument. After that, we suggest to (1) prioritise the update of high-quality CGs or (2) improve the methodological quality of the CG during the updating process. After the updating process, CG developers can assess the reporting of the updating process using CheckUp. Consequently, when both the AGREE II and CheckUp instruments are properly applied, developers will have a complete and detailed overview of the quality of the developing and updating processes. Afterwards, if applicable, the prioritisation process of updating CGs can be conducted [11].

There is currently no gold standard for updating CGs [18, 19, 94, 95]. Although CheckUp does not evaluate the quality of the updating process, CG developers can use it to inform their updating processes. Additionally, CheckUp can be used by interested CG users to assess whether updated CGs are in alignment with the CheckUp items, and editors of scientific journals that publish updated CGs may request the completion of CheckUp from the CG authors [20].

It would be relevant to monitor the use and the impact of CheckUp in the updating CG field over the next few years, potentially using this study as a baseline evaluation before the publication of CheckUp. Finally, we invite users to comment on the items and the usability of CheckUp contacting the corresponding author of this publication.

We suggest users of CheckUp to assess the reporting of the updating process in updated CGs by at least three calibrate reviewers. We involved three reviewers for convenience to avoid ties. Further examinations of CheckUp are required to determine if the inter-observer agreement between two reviewers would be adequate. Clinical expertise regarding the clinical area of the CG is not required; however, methodological comprehension on the updating process of CGs is highly desirable. To facilitate understanding of the domain scores and overall scores, we have transformed the domain and overall scores to a 10-point scale score.

## Conclusions

The reporting of the updating process in updated CGs is suboptimal. Presentation of updated CGs and the methodology of the updating process where areas where more work is needed. We advise CG developers to use CheckUp to improve the reporting of updated CGs. CheckUp can also be used to assess the updating process in updated CGs and as a blueprint that could be used to inform specific updating methods and reporting strategies.

## Additional files

Additional file 1: Literature search strategy. (DOCX 27 kb)

Additional file 2: Excluded full text references including reason for exclusion. (DOCX 52 kb)

## Abbreviations

AGREE: Appraisal of Guidelines for Research and Evaluation
CG: Clinical guideline
CheckUp: Checklist for the Reporting of Updated Guidelines
CI: Confidence interval
G-I-N: Guidelines International Network
ICC: Interclass Correlations Coefficient
