# Correction to: Updated clinical guidelines experience major reporting limitations

**DOI:** 10.1186/s13012-018-0759-0

**Published:** 2018-04-30

**Authors:** Robin W. M. Vernooij, Laura Martínez García, Ivan Dario Florez, Laura Hidalgo Armas, Michiel H. F. Poorthuis, Melissa Brouwers, Pablo Alonso-Coello

**Affiliations:** 1 Iberoamerican Cochrane Centre, Biomedical Research Institute Sant Pau (IIB Sant Pau), Barcelona, Spain; 2 Department of Health Research Methods, Evidence and Impact, McMaster University, Hamilton, Canada; 3 Department of Pediatrics, University of Antioquia, Medellin, Colombia; 4 University Medical Center Utrecht, Utrecht, The Netherlands; 5 Department of Oncology, McMaster University, Hamilton, Canada; 6 CIBER of Epidemiology and Public Health (CIBERESP), Madrid, Spain

## Correction

After publication of the original article [1] it was brought to our attention that author Laura Hidalgo Armas was incorrectly included as Laura Hildago Armas.

The correct spelling of the name is included in the author list of this ‘Correction’.
