# Supplementary material for: Updated clinical guidelines experience major reporting limitations
Source: Implement Sci. 2017 Oct 12;12:120. doi: 10.1186/s13012-017-0651-3 (PMC5639761; doi:10.1186/s13012-017-0651-3)
Supplement: Supplementary file 1 — Literature search strategy. (DOCX 27 kb) [file 13012_2017_651_MOESM1_ESM.docx]

**Appendix I**

**Ovid MEDLINE(R) In-Process & Other Non-Indexed Citations, Ovid MEDLINE(R) Daily and Ovid MEDLINE(R) 1946 to Present (accessed through OVID)** – 04-08-2016

| **Search** | **Query** | **Items found** |
| --- | --- | --- |
| [#13](http://www.ncbi.nlm.nih.gov/pubmed/advanced) | **limit 12 to (yr="2015" and english)** | 1313 |
| [#12](http://www.ncbi.nlm.nih.gov/pubmed/advanced) | **6 AND 11** | 12821 |
| [#10](http://www.ncbi.nlm.nih.gov/pubmed/advanced) | **7 OR 8 OR 9** | 124541 |
| #9 | **Actuali*.ti,ab.** | 2363 |
| [#8](http://www.ncbi.nlm.nih.gov/pubmed/advanced) | **Up to date.ti,ab.** | 15603 |
| [#7](http://www.ncbi.nlm.nih.gov/pubmed/advanced) | **Updat*.ti,ab.** | 108135 |
| [#6](http://www.ncbi.nlm.nih.gov/pubmed/advanced) | **1 OR 2 OR 3 OR 4 OR 5** | 299085 |
| [#5](http://www.ncbi.nlm.nih.gov/pubmed/advanced) | **Practice Guideline/** | 21695 |
| [#4](http://www.ncbi.nlm.nih.gov/pubmed/advanced) | **Practice Guidelines as Topic/** | 94116 |
| [#3](http://www.ncbi.nlm.nih.gov/pubmed/advanced) | **Guideline*.ti,ab.** | 238096 |
| #2 | **Clinical guideline*.ti,ab.** | 8707 |
| #1 | **Clinical Practice Guideline*.ti,ab.** | [9537](http://www.ncbi.nlm.nih.gov/pubmed/?cmd=HistorySearch&querykey=10) |

**G-I-N (**[**http://www.g-i-n.net/**](http://www.g-i-n.net/)**)** – 04-08-2016

1. Language: English
2. Publication: Guideline

We found 1173 results out of 6237 entries for your search.

1. Select guidelines published in 2015

82 results

**National Guidelines Clearinghouse (**[**http://www.guideline.gov/search/advanced-search.aspx**](http://www.guideline.gov/search/advanced-search.aspx)**) –** 04-08-2016

1. Publication Year: 2015

165 results
