# Supplementary material for: Updated clinical guidelines experience major reporting limitations
Source: Implement Sci. 2017 Oct 12;12:120. doi: 10.1186/s13012-017-0651-3 (PMC5639761; doi:10.1186/s13012-017-0651-3)
Supplement: Supplementary file 2 — Excluded full text references including reason for exclusion. (DOCX 52 kb) [file 13012_2017_651_MOESM2_ESM.docx]

| **#** | **Reference** | **Reason for exclusion** |
| --- | --- | --- |
| #1 | America IIDSo. A Compendium of Strategies to Prevent Healthcare-Associated Infections in Acute Care Hospitals. 2015 [cited; Available from: http://www.jstor.org/stable/10.1086/593984 | Not published in 2015 |
| #2 | America IIDSo. Community-Acquired Pneumonia in Adults: Guidelines for Management. 2015 [cited; Available from: http://cid.oxfordjournals.org/content/44/Supplement_2/S27.full | Not published in 2015 |
| #3 | America IIDSo. Diagnosis and Management of Bone and Joint Infections in Children. 2015 [cited; Available from: http://www.idsociety.org/Organ_System/ | Guideline with no systematic review |
| #4 | America IIDSo. IDSA/SHEA Clinical Practice Guidelines on Antimicrobial Stewardship. 2015 [cited; Available from: http://www.idsociety.org/Antimicrobial_Agents/ | Not published in 2015 |
| #5 | America IIDSo. Practice Guidelines for Outpatient Parenteral Antimicrobial Therapy. 2015 [cited; Available from: http://cid.oxfordjournals.org/content/38/12/1651.full | Not published in 2015 |
| #6 | America IIDSo. Practice Guidelines for the Management of Bacterial Meningitis. 2015 [cited; Available from: http://cid.oxfordjournals.org/content/39/9/1267.full | Not published in 2015 |
| #7 | America IIDSo. Seasonal Influenza in Adults and Children—Diagnosis, Treatment, Chemoprophylaxis, and Institutional Outbreak Management: Clinical Practice Guidelines of the Infectious Diseases Society of America. 2015 [cited; Available from: <http://cid.oxfordjournals.org/content/48/8/1003.1.full> | Not published in 2015 |
| #8 | America IIDSo. Treatment of Aspergillosis. 2015 [cited; Available from: <http://www.idsociety.org/uploadedFiles/IDSA/Guidelines-Patient_Care/PDF_Library/Aspergillosis.pdf> | No updated guideline |
| #9 | America IIDSo. Vancomycin Therapeutic Monitoring: Review and Recommendations from the ASHP, IDSA and SIDP Task Force. 2015 [cited; Available from: http://www.ajhp.org/content/66/1/82.full | Not published in 2015 |
| #10 | America IIDSo. Clinical Practice Guidelines for Clostridium difficile Infection in Adults: 2010 Update by the Society for Healthcare Epidemiology of America (SHEA) and the Infectious Diseases Society of America (IDSA). 2015 [cited; Available from: http://www.jstor.org/stable/10.1086/651706 | Not published in 2015 |
| #11 | American Academy of Neurology / American Association of N, Electrodiagnostic M. Evidence-based guideline summary: evaluation, diagnosis, and management of facioscapulohumeral muscular dystrophy: report of the Guideline Development, Dissemination, and Implementation Subcommittee of the American Academy of Neurology and the Practice Issues Review Panel of the American Association of Neuromuscular &amp; Electrodiagnostic Medicine. 2015. | No updated guideline |
| #12 | American Academy of Neurology / American Association of N, Electrodiagnostic M. Evidence-based guideline summary: evaluation, diagnosis, and management of congenital muscular dystrophy: report of the Guideline Development Subcommittee of the American Academy of Neurology and the Practice Issues Review Panel of the American Association of Neuromuscular and Electrodiagnostic Medicine. 2015. | No updated guideline |
| #13 | American College of P. Treatment of pressure ulcers: a clinical practice guideline from the American College of Physicians. 2015. | No updated guideline |
| #14 | American Dental A. Evidence-based clinical practice guideline on the nonsurgical treatment of chronic periodontitis by means of scaling and root planing with or without adjuncts. 2015. | No updated guideline |
| #15 | American Diabetes A. (11) Children and adolescents. Diabetes Care. 2015; 38 Suppl: S70-6. http://dx.doi.org/10.2337/dc15-S014. | Guideline with no systematic review |
| #16 | American Occupational Therapy Association I. Occupational therapy practice guidelines for adults with stroke. 2008. | No updated guideline |
| #17 | Argoff CE, Brennan MJ, Camilleri M, et al. Consensus Recommendations on Initiating Prescription Therapies for Opioid-Induced Constipation. Pain Med. 2015; 16: 2324-37. http://dx.doi.org/10.1111/pme.12937. | No updated guideline |
| #18 | Association of periOperative Registered N. Guideline for prevention of unplanned patient hypothermia. 2015. | Guideline without recommendation |
| #19 | Atkins DL, Berger S, Duff JP, et al. Part 11: Pediatric Basic Life Support and Cardiopulmonary Resuscitation Quality: 2015 American Heart Association Guidelines Update for Cardiopulmonary Resuscitation and Emergency Cardiovascular Care. Circulation. 2015; 132: S519-25. <http://dx.doi.org/10.1161/CIR.0000000000000265>. | Duplicate |
| #20 | Atkins DL, Berger S, Duff JP, et al. Part 11: Pediatric Basic Life Support and Cardiopulmonary Resuscitation Quality: 2015 American Heart Association Guidelines Update for Cardiopulmonary Resuscitation and Emergency Cardiovascular Care. Circulation. 2015; 132: S519-25. <http://dx.doi.org/10.1161/CIR.0000000000000265>. | Duplicate |
| #21 | Australia CCACC. Clinical Practice Guidelines for the Management of Sarcoma in AYA. 2015 [cited; Available from: <http://wiki>.cancer.org.au/ackling/Guidelines:Sarcoma/AYA | Not published in 2015 |
| #22 | Berger AR, Cruess AF, Altomare F, et al. Optimal Treatment of Retinal Vein Occlusion: Canadian Expert Consensus. Ophthalmologica. 2015; 234: 6-25. <http://dx>.doi.org/10.1159/000381357. | No updated guideline |
| #23 | Binepal N, Lemyre B, Dunn S, et al. Systematic Review and Quality Appraisal of International Guidelines on Perinatal Care of Extremely Premature Infants. 2015; 11: 126-34. | Guideline without recommendation |
| #24 | Birken SA, Ellis SD, Walker JS, et al. Guidelines for the use of survivorship care plans: a systematic quality appraisal using the AGREE II instrument. Implement Sci. 2015; 10: 63. <http://dx>.doi.org/10.1186/s13012-015-0254-9. | No updated guideline |
| #25 | Bossaert LL, Perkins GD, Askitopoulou H, et al. European Resuscitation Council Guidelines for Resuscitation 2015: Section 11. The ethics of resuscitation and end-of-life decisions. Resuscitation. 2015; 95: 302-11. <http://dx>.doi.org/10.1016/j.resuscitation.2015.07.033. | Guideline with no systematic review |
| #26 | Brackstone M, Fletcher GG, Dayes IS, et al. Locoregional therapy of locally advanced breast cancer: a clinical practice guideline. Curr. 2015; 22: S54-66. <http://dx>.doi.org/10.3747/co.22.2316. | No updated guideline |
| #27 | British Thoracic S. British Thoracic Society guidelines for home oxygen use in adults. 2015. | No updated guideline |
| #28 | British Thoracic S. British Thoracic Society guidelines for the investigation and management of pulmonary nodules. 2015. | No updated guideline |
| #29 | Canadian Task Force on Preventive Health C. Recommendations for growth monitoring, and prevention and management of overweight and obesity in children and youth in primary care. 2015. | No updated guideline |
| #30 | Casani AP, Dallan I, Navari E, et al. Vertigo in childhood: proposal for a diagnostic algorithm based upon clinical experience. Acta Otorhinolaryngol Ital. 2015; 35: 180-5. | Guideline with no systematic review |
| #31 | Centre KCEBHK. Renal cancer in adults: diagnosis, treatment and follow-up. 2015 [cited; Available from: <http://kce>.fgov.be/publication/report/renal-cancer-in-adults-diagnosis-treatment-and-follow-up | No updated guideline |
| #32 | Centre KCEBHK. Oncogenetic testing and follow-up for women with familial breast/ovarian cancer, Li Fraumeni syndrome and Cowden syndrome. 2015 [cited; Available from: <https://kce>.fgov.be/node/2674/ | No updated guideline |
| #33 | Centre KCEBHK. Oncogenetic testing, diagnosis and follow-up in Birt-Hogg-Dubé syndrome, familial atypical multiple mole melanoma syndrome and neurofibromatosis 1 and 2. 2015 [cited; Available from: <http://kce>.fgov.be/node/2810/ | No updated guideline |
| #34 | Centre KCEBHK. Oncogenetic testing for persons with hereditary endocrine cancer syndromes. 2015 [cited; Available from: <http://kce>.fgov.be/node/2817/ | No updated guideline |
| #35 | Chan RH, Shaw JL, Hauser TH, et al. Guideline Adherence for Echocardiographic Follow-Up in Outpatients with at Least Moderate Valvular Disease. J Am Soc Echocardiogr. 2015; 28: 795-801. <http://dx>.doi.org/10.1016/j.echo.2015.03.001. | Guideline with no systematic review |
| #36 | Cheson BD. Staging and response assessment in lymphomas: the new Lugano classification. Chin. 2015; 4: 5. <http://dx>.doi.org/10.3978/j.issn.2304-3865.2014.11.03. | Guideline with no systematic review |
| #37 | Clark JR, Scott SD, Jack AL, et al. Monitoring of chimerism following allogeneic haematopoietic stem cell transplantation (HSCT): technical recommendations for the use of short tandem repeat (STR) based techniques, on behalf of the United Kingdom National External Quality Assessment Service for Leucocyte Immunophenotyping Chimerism Working Group. Br J Haematol. 2015; 168: 26-37. <http://dx>.doi.org/10.1111/bjh.13073. | No updated guideline |
| #38 | College of American Pathologists / National Society for H. Uniform ackling of blocks and slides in surgical pathology: guideline from the College of American Pathologists Pathology and Laboratory Quality Center and the National Society for Histotechnology. 2015. | No updated guideline |
| #39 | Crowley JJ, Weinberg JM, Wu JJ, et al. Treatment of nail psoriasis: best practice recommendations from the Medical Board of the National Psoriasis Foundation. JAMA Dermatol. 2015; 151: 87-94. <http://dx>.doi.org/10.1001/jamadermatol.2014.2983. | No updated guideline |
| #40 | Dalton HJ, Macrae DJ, Pediatric Acute Lung Injury Consensus Conference G. Extracorporeal support in children with pediatric acute respiratory distress syndrome: proceedings from the Pediatric Acute Lung Injury Consensus Conference. Pediatr Crit Care Med. 2015; 16: S111-7. <http://dx>.doi.org/10.1097/PCC.0000000000000439. | No updated guideline |
| #41 | Daniels SR. Pediatric guidelines for dyslipidemia. J. 2015; 9: S5-S10. <http://dx>.doi.org/10.1016/j.jacl.2015.03.105. | Guideline with no systematic review |
| #42 | Darlow B, Campbell N, Austin N, et al. The prevention of early-onset neonatal group B streptococcus infection: New Zealand Consensus Guidelines 2014. N Z Med J. 2015; 128: 69-76. | No updated guideline |
| #43 | Estes NA, 3rd, Kovacs RJ, Baggish AL, Myerburg RJ. Eligibility and Disqualification Recommendations for Competitive Athletes With Cardiovascular Abnormalities: Task Force 11: Drugs and Performance-Enhancing Substances: A Scientific Statement From the American Heart Association and American College of Cardiology. J Am Coll Cardiol. 2015; 66: 2429-33. <http://dx>.doi.org/10.1016/j.jacc.2015.09.043. | No updated guideline |
| #44 | Estes NA, 3rd, Kovacs RJ, Baggish AL, et al. Eligibility and Disqualification Recommendations for Competitive Athletes With Cardiovascular Abnormalities: Task Force 11: Drugs and Performance-Enhancing Substances: A Scientific Statement From the American Heart Association and American College of Cardiology. Circulation. 2015; 132: e330-3. <http://dx>.doi.org/10.1161/CIR.0000000000000247. | No updated guideline |
| #45 | European Academy of N. EFNS-ENS/EAN guideline on concomitant use of cholinesterase inhibitors and memantine in moderate to severe Alzheimer’s disease. 2015 | No updated guideline |
| #46 | Excellence NNIfHC. Bronchiolitis (CG). 2015 [cited; Available from: <http://guidance>.nice.org.uk/CG/WaveR/136 | No updated guideline |
| #47 | Excellence NNIfHC. Asthma (CG). 2015 [cited; Available from: <http://guidance>.nice.org.uk/CG/Wave0/640 | No updated guideline |
| #48 | Excellence NNIfHC. Challenging behaviour in people with learning disability (CG). 2015 [cited; Available from: <http://guidance>.nice.org.uk/CG/Wave0/654 | No updated guideline |
| #49 | Excellence NNIfHC. Children’s attachment (CG). 2015 [cited; Available from: <http://guidance>.nice.org.uk/CG/Wave0/675 | No updated guideline |
| #50 | Excellence NNIfHC. Bladder cancer (CG). 2015 [cited; Available from: <http://guidance>.nice.org.uk/CG/Wave0/600 | No updated guideline |
| #51 | Excellence NNIfHC. Excess winter deaths and illnesses (PH). 2015 [cited; Available from: <http://guidance>.nice.org.uk/PHG/70 | Not published in 2015 |
| #52 | Excellence NNIfHC. Gallstone disease (CG). 2015 [cited; Available from: <http://guidance>.nice.org.uk/CG/Wave0/657 | No updated guideline |
| #53 | Excellence NNIfHC. Disability, dementia and frailty in later life – mid-life approaches to prevention (PH). 2015 [cited; Available from: <http://guidance>.nice.org.uk/PHG/64 | No updated guideline |
| #54 | Excellence NNIfHC. Gastro-oesophageal reflux in children and young people (CG). 2015 [cited; Available from: <http://guidance>.nice.org.uk/CG/Wave0/599 | No updated guideline |
| #55 | Excellence NNIfHC. Older people: independence and mental wellbeing (PH). 2015 [cited; Available from: <http://guidance>.nice.org.uk/PHG/65 | Guideline with no systematic review |
| #56 | Excellence NNIfHC. Intravenous fluids therapy in children (CG). 2015 [cited; Available from: <http://guidance>.nice.org.uk/CG/Wave0/655 | No updated guideline |
| #57 | Excellence NNIfHC. Macular degeneration (CG). 2015 [cited; Available from: <http://guidance>.nice.org.uk/CG/Wave0/658 | Guideline with no systematic review |
| #58 | Excellence NNIfHC. Maintaining a healthy weight and preventing excess weight gain among children and adults (PH). 2015 [cited; Available from: <http://guidance>.nice.org.uk/PHG/78 | Guideline with no systematic review |
| #59 | Excellence NNIfHC. Major trauma services (CG). 2015 [cited; Available from: <http://guidance>.nice.org.uk/CG/Wave0/641 | Not published in 2015 |
| #60 | Excellence NNIfHC. Melanoma (CG). 2015 [cited; Available from: <http://guidance>.nice.org.uk/index.jsp?action=byId&o=13771 | No updated guideline |
| #61 | Excellence NNIfHC. Menopause (CG). 2015 [cited; Available from: <http://guidance>.nice.org.uk/CG/Wave0/639 | No updated guideline |
| #62 | Excellence NNIfHC. Oral health promotion approaches for dental health practitioners (PH). 2015 [cited; Available from: <http://guidance>.nice.org.uk/PHG/60 | Guideline with no systematic review |
| #63 | Excellence NNIfHC. Non-Hodgkin’s lymphoma (CG). 2015 [cited; Available from: <http://guidance>.nice.org.uk/CG/Wave0/671 | Not published in 2015 |
| #64 | Excellence NNIfHC. Promoting oral health – the patient experience (PH). 2015 [cited; Available from: <http://guidance>.nice.org.uk/PHG/60 | Not published in 2015 |
| #65 | Excellence NNIfHC. Sunlight exposure: benefits and safety (PH). 2015 [cited; Available from: <http://guidance>.nice.org.uk/index.jsp?action=byId&o=13796 | Not published in 2015 |
| #66 | Excellence NNIfHC. Transfusion (CG). 2015 [cited; Available from: <http://guidance>.nice.org.uk/CG/Wave0/663 | No updated guideline |
| #67 | Excellence NNIfHC. Workplace policy and management practices to improve the health and wellbeing of employees (PH). 2015 [cited; Available from: <http://guidance>.nice.org.uk/PHG/57 | No updated guideline |
| #68 | Excellence NNIfHC. Upper airways tract cancers (CG). 2015 [cited; Available from: <http://guidance>.nice.org.uk/CG/Wave0/668 | Not published in 2015 |
| #69 | Excellence NNIfHC. Diabetic foot problems - inpatient management (CG119). 2015 [cited; Available from: http://guidance.nice.org.uk/CG119 | Duplicate |
| #70 | Excellence NNIfHC. Type 2 Diabetes - newer agents (partial update of CG66) (CG87). 2015 [cited; Available from: http://guidance.nice.org.uk/CG87 | Duplicate |
| #71 | Fleming N, O'Driscoll T, Becker G, et al. Adolescent Pregnancy Guidelines. J Obstet Gynaecol Can. 2015; 37: 740-59. | No updated guideline |
| #72 | Force USPST. Screening for thyroid dysfunction: U.S. Preventive Services Task Force recommendation statement. 1996. | Guideline with no systematic review |
| #73 | Force USPST. Screening for speech and language delay and disorders in children aged 5 years or younger: U.S. Preventive Services Task Force recommendation statement. 2006. | Guideline with no systematic review |
| #74 | Foundation AHAAoO-HNS. Clinical Practice Guideline: Allergic Rhinitis. 2015 [cited; Available from: http://www.entnet.org/Practice/clinicalPracticeguidelines.cfm | No updated guideline |
| #75 | Foundation AHAAoO-HNS. Clinical practice guideline: Adult sinusitis. 2015 [cited; Available from: <http://oto.sagepub.com/content/152/2_suppl> | Duplicate |
| #76 | French CT, Diekemper RL, Irwin RS, Chest Expert Cough P. Assessment of Intervention Fidelity and Recommendations for Researchers Conducting Studies on the Diagnosis and Treatment of Chronic Cough in the Adult: CHEST Guideline and Expert Panel Report. Chest. 2015; 148: 32-54. http://dx.doi.org/10.1378/chest.15-0164. | No updated guideline |
| #77 | French CT, Diekemper RL, Irwin RS, Chest Expert Cough P. Assessment of Intervention Fidelity and Recommendations for Researchers Conducting Studies on the Diagnosis and Treatment of Chronic Cough in the Adult: CHEST Guideline and Expert Panel Report. Chest. 2015; 148: 32-54. http://dx.doi.org/10.1378/chest.15-0164. | Duplicate |
| #78 | Halperin JJ, Kurlan R, Schwalb JM, et al. Practice guideline: Idiopathic normal pressure hydrocephalus: Response to shunting and predictors of response: Report of the Guideline Development, Dissemination, and Implementation Subcommittee of the American Academy of Neurology. Neurology. 2015; 85: 2063-71. http://dx.doi.org/10.1212/WNL.0000000000002193. | No updated guideline |
| #79 | Halperin JJ, Kurlan R, Schwalb JM, et al. Practice guideline: Idiopathic normal pressure hydrocephalus: Response to shunting and predictors of response: Report of the Guideline Development, Dissemination, and Implementation Subcommittee of the American Academy of Neurology. Neurology. 2015; 85: 2063-71. http://dx.doi.org/10.1212/WNL.0000000000002193. | Duplicate |
| #80 | Han H, Chao H, Guerra A, et al. Evolution of the American College of Cardiology/American Heart Association Clinical Guidelines. J Am Coll Cardiol. 2015; 65: 2726-34. http://dx.doi.org/10.1016/j.jacc.2015.04.050. | Guideline without recommendation |
| #81 | Hoffmeister A, Mayerle J, Beglinger C, et al. English language version of the S3-consensus guidelines on chronic pancreatitis: Definition, aetiology, diagnostic examinations, medical, endoscopic and surgical management of chronic pancreatitis. Z Gastroenterol. 2015; 53: 1447-95. http://dx.doi.org/10.1055/s-0041-107379. | No updated guideline |
| #82 | Horner K, O'Malley L, Taylor K, Glenny AM. Guidelines for clinical use of CBCT: a review. Dentomaxillofac Radiol. 2015; 44: 20140225. http://dx.doi.org/10.1259/dmfr.20140225. | No updated guideline |
| #83 | Horner K, O'Malley L, Taylor K, Glenny AM. Guidelines for clinical use of CBCT: a review. Dentomaxillofac Radiol. 2015; 44: 20140225. http://dx.doi.org/10.1259/dmfr.20140225. | Duplicate |
| #84 | Hta DoH - Hta Unit MoHM. Management of Multiple Sclerosis. 2015 [cited; Available from: http://www.moh.gov.my/penerbitan/CPG/CPG%20Management%20of%20Multiple%20Sclerosis.compressed.pdf | No updated guideline |
| #85 | Iepsen UW, Jorgensen KJ, Ringbaek T, et al. A combination of resistance and endurance training increases leg muscle strength in COPD: An evidence-based recommendation based on systematic review with meta-analyses. Chron. 2015; 12: 132-45. http://dx.doi.org/10.1177/1479972315575318. | No updated guideline |
| #86 | Jain V, Chari R, Maslovitz S, et al. Guidelines for the Management of a Pregnant Trauma Patient. J Obstet Gynaecol Can. 2015; 37: 553-74. | No updated guideline |
| #87 | Kennedy A, Bester L, Salem R, et al. Role of hepatic intra-arterial therapies in metastatic neuroendocrine tumours (NET): guidelines from the NET-Liver-Metastases Consensus Conference. Hpb. 2015; 17: 29-37. http://dx.doi.org/10.1111/hpb.12326. | No updated guideline |
| #88 | Li CC, Wang YQ, Li YP, Li XL. Critical appraisal of clinical practice guidelines for treating pancreatic cancer based on the global disease burden. J Evid Based Med. 2015; 8: 11-21. http://dx.doi.org/10.1111/jebm.12140. | Guideline with no systematic review |
| #89 | Liang B, Lai JM, Murugan A, et al. Proposed Guidelines for Treatment of Concomitant Distal Radius and Distal Ulna Fractures. Hand Surg. 2015; 20: 396-401. http://dx.doi.org/10.1142/S0218810415500306. | Guideline with no systematic review |
| #90 | Llort G, Chirivella I, Morales R, et al. SEOM clinical guidelines in Hereditary Breast and ovarian cancer. Clin Transl Oncol. 2015; 17: 956-61. http://dx.doi.org/10.1007/s12094-015-1435-3. | Guideline with no systematic review |
| #91 | Ltd TGLTG. Therapeutic Guidelines: Dermatology. 2015 [cited; Available from: http://www.tg.com.au/?sectionid=43 | No updated guideline |
| #92 | Mandl P, Navarro-Compan V, Terslev L, et al. EULAR recommendations for the use of imaging in the diagnosis and management of spondyloarthritis in clinical practice. Ann Rheum Dis. 2015; 74: 1327-39. http://dx.doi.org/10.1136/annrheumdis-2014-206971. | No updated guideline |
| #93 | Mandl P, Navarro-Compan V, Terslev L, et al. EULAR recommendations for the use of imaging in the diagnosis and management of spondyloarthritis in clinical practice. Ann Rheum Dis. 2015; 74: 1327-39. http://dx.doi.org/10.1136/annrheumdis-2014-206971. | Duplicate |
| #94 | McCulloch KL, Goldman S, Lowe L, et al. Development of clinical recommendations for progressive return to activity after military mild traumatic brain injury: guidance for rehabilitation providers. J Head Trauma Rehabil. 2015; 30: 56-67. http://dx.doi.org/10.1097/HTR.0000000000000104. | No updated guideline |
| #95 | McQuilten ZK, Crighton G, Engelbrecht S, et al. Transfusion interventions in critical bleeding requiring massive transfusion: a systematic review. Transfus Med Rev. 2015; 29: 127-37. http://dx.doi.org/10.1016/j.tmrv.2015.01.001. | No updated guideline |
| #96 | Medicine KKIoO. Evidence-Based Clinical Practice Guidelines for Ankle Sprain in Adults on Traditional Korean Medicine. 2015 [cited; Available from: https://www.kiom.re.kr/brdartcl/boardarticleList.do?menu_nix=WUNNW2Aq&brd_id=BDIDX_o9YEVvNb40b134N1Rt17aq&searchKeyword=%EC%9E%84%EC%83%81%EC%A7%84%EB%A3%8C%EC%A7%80%EC%B9%A8&searchCondition=SC_ALL | Not published in English |
| #97 | Medicine KKIoO. Evidence-Based Clinical Practice Guidelines for Idiopathic Facial Paralysus in Adults on Traditional Korean Medicine. 2015 [cited; Available from: https://www.kiom.re.kr/brdartcl/boardarticleList.do?menu_nix=WUNNW2Aq&brd_id=BDIDX_o9YEVvNb40b134N1Rt17aq&searchKeyword=%EC%9E%84%EC%83%81%EC%A7%84%EB%A3%8C%EC%A7%80%EC%B9%A8&searchCondition=SC_ALL | Not published in English |
| #98 | Medicine KKIoO. Evidence-Based Clinical Practice Guidelines for Shoulder pain on Traditional Korean Medicine. 2015 [cited; Available from: https://www.kiom.re.kr/brdartcl/boardarticleList.do?menu_nix=WUNNW2Aq&brd_id=BDIDX_o9YEVvNb40b134N1Rt17aq&searchKeyword=%EC%9E%84%EC%83%81%EC%A7%84%EB%A3%8C%EC%A7%80%EC%B9%A8&searchCondition=SC_ALL | Not published in English |
| #99 | Miyazaki M, Yoshitomi H, Miyakawa S, et al. Clinical practice guidelines for the management of biliary tract cancers 2015: the 2nd English edition. J Hepatobiliary Pancreat Sci. 2015; 22: 249-73. <http://dx.doi.org/10.1002/jhbp.233>. | Duplicate |
| #100 | Mostl K, Addie DD, Boucraut-Baralon C, et al. Something old, something new: Update of the 2009 and 2013 ABCD guidelines on prevention and management of feline infectious diseases. J Feline Med Surg. 2015; 17: 570-82. http://dx.doi.org/10.1177/1098612X15588448. | No updated guideline |
| #101 | Mostl K, Addie DD, Boucraut-Baralon C, et al. Something old, something new: Update of the 2009 and 2013 ABCD guidelines on prevention and management of feline infectious diseases. J Feline Med Surg. 2015; 17: 570-82. http://dx.doi.org/10.1177/1098612X15588448. | Duplicate |
| #102 | Munasinghe LL, Willows N, Yuan Y, Veugelers PJ. Dietary reference intakes for vitamin D based on the revised 2010 dietary guidelines are not being met by children in Alberta, Canada. Nutr Res. 2015; 35: 956-64. http://dx.doi.org/10.1016/j.nutres.2015.07.006. | No updated guideline |
| #103 | Nahirniak S, Slichter SJ, Tanael S, et al. Guidance on platelet transfusion for patients with hypoproliferative thrombocytopenia. Transfus Med Rev. 2015; 29: 3-13. http://dx.doi.org/10.1016/j.tmrv.2014.11.004. | No updated guideline |
| #104 | National Clinical Guideline C. Type 1 diabetes in adults: diagnosis and management. 2004. | Duplicate |
| #105 | National Clinical Guideline Centre for A, Chronic C. Venous thromboembolism in adults admitted to hospital: reducing the risk. 2007. | Not published in 2015 |
| #106 | National Clinical Guideline Centre for A, Chronic C. Lower urinary tract symptoms in men: assessment and management. 2010. | Not published in 2015 |
| #107 | National Collaborating Centre for C. Suspected cancer: recognition and referral. 2005. | Duplicate |
| #108 | National Collaborating Centre for C. Melanoma: assessment and management. 2015. | No updated guideline |
| #109 | National Collaborating Centre for Mental H. Depression in children and young people: identification and management in primary, community and secondary care. 2005. | Not published in 2015 |
| #110 | National Collaborating Centre for Mental H. Violence and aggression: short-term management in mental health, health and community settings. 2005. | Duplicate |
| #111 | National Collaborating Centre for Ws, Children's H. Diabetes in pregnancy: management of diabetes and its complications from preconception to the postnatal period. 2008. | Duplicate |
| #112 | National Institute for H, Care E. Infliximab, adalimumab and golimumab for treating moderately to severely active ulcerative colitis after the failure of conventional therapy (including a review of TA140 and TA262). 2008. | Guideline with no systematic review |
| #113 | National Institute for H, Care E. Coeliac disease: recognition, assessment and management. 2009. | Duplicate |
| #114 | National Institute for H, Care E. Diabetic foot problems: prevention and management. 2011. | Duplicate |
| #115 | National Institute for H, Care E. Erlotinib and gefitinib for treating non-small-cell lung cancer that has progressed after prior chemotherapy. 2015. | Guideline with no systematic review |
| #116 | National Institute for H, Care E. Naloxegol for treating opioid-induced constipation. 2015. | Guideline with no systematic review |
| #117 | National Institute for H, Care E. Older people with social care needs and multiple long-term conditions. 2015. | No updated guideline |
| #118 | National Institute for H, Care E. Apremilast for treating moderate to severe plaque psoriasis. 2015. | Guideline with no systematic review |
| #119 | National Institute for H, Care E. Type 2 diabetes in adults: management. 2015. | Duplicate |
| #120 | Ned-Sykes R, Johnson C, Ridderhof JC, et al. Competency Guidelines for Public Health Laboratory Professionals: CDC and the Association of Public Health Laboratories. Morb Mortal Wkly Rep Surveill Summ. 2015; 64 Suppl 1: 1-81. | Guideline with no systematic review |
| #121 | Ned-Sykes R, Johnson C, Ridderhof JC, et al. Competency Guidelines for Public Health Laboratory Professionals: CDC and the Association of Public Health Laboratories. MMWR (Suppl). 2015; 64: 1-81. | Guideline with no systematic review |
| #122 | Network SSIG. Glaucoma referral and safe discharge (SIGN CPG 144). 2015 [cited; Available from: <http://www.sign.ac.uk/guidelines/fulltext/144/index.html> | No updated guideline |
| #123 | Network SSIG. Management of osteoporosis and the prevention of fragility fractures (SIGN CPG 142). 2015 [cited; Available from: http://www.sign.ac.uk/guidelines/fulltext/142/index.html | No updated guideline |
| #124 | Oncology AASoC. Definitive and Adjuvant Radiotherapy in Locally Advanced Non–Small-Cell Lung Cancer: American Society of Clinical Oncology Clinical Practice Guideline Endorsement of the American Society for Radiation Oncology Evidence-Based Clinical Practice Guideline. 2015 [cited; Available from: http://www.instituteforquality.org/definitive-and-adjuvant-radiotherapy-locally-advanced-non%E2%80%93small-cell-lung-cancer-american-society | No updated guideline |
| #125 | Oncology AASoC. Recommendations for the Use of WBC Growth Factors: American Society of Clinical Oncology Clinical Practice Guideline Update. 2015 [cited; Available from: <http://www.instituteforquality.org/asco-2006-update-recommendations-use-white-blood-cell-growth-factors-evidence-based-clinical> | Duplicate |
| #126 | ASCO - American Society of Clinical Oncology. Prostate Cancer Survivorship Care Guideline: American Society of Clinical Oncology Clinical Practice Guideline Endorsement. [Guideline] 2015 [cited; Available from: http://www.instituteforquality.org/prostate-cancer-survivorship-care-guideline-american-society-clinical-oncology-clinical-practice | No updated guideline |
| #127 | Oncology AASoC. Postoperative Radiation Therapy for Endometrial Cancer: American Society of Clinical Oncology Clinical Practice Guideline Endorsement of the American Society for Radiation Oncology Evidence-Based Guideline. 2015 [cited; Available from: <http://www.instituteforquality.org/postoperative-radiation-therapy-endometrial-cancer-american-society-clinical-oncology-clinical> | No updated guideline |
| #128 | Oncology AASoC. Treatment of Small-Cell Lung Cancer: American Society of Clinical Oncology Endorsement of the American College of Chest Physicians Guideline. 2015 [cited; Available from: http://www.instituteforquality.org/treatment-small-cell-lung-cancer-american-society-clinical-oncology-endorsement-american-college | No updated guideline |
| #129 | Oncology AASoC. Systemic Therapy for Stage IV Non–Small-Cell Lung Cancer: American Society of Clinical Oncology Clinical Practice Guideline Update. 2015 [cited; Available from: http://www.instituteforquality.org/asco-clinical-practice-guideline-update-chemotherapy-stage-iv-non-small-cell-lung-cancer | Duplicate |
| #130 | Oncology AASoC. Use of Biomarkers to Guide Decisions on Systemic Therapy for Women With Metastatic Breast Cancer: American Society of Clinical Oncology Clinical Practice Guideline. 2015 [cited; Available from: http://www.instituteforquality.org/use-biomarkers-guide-decisions-systemic-therapy-women-metastatic-breast-cancer-american-society | Duplicate |
| #131 | Patel MB, Humble SS, Cullinane DC, et al. Cervical spine collar clearance in the obtunded adult blunt trauma patient: a systematic review and practice management guideline from the Eastern Association for the Surgery of Trauma. J Trauma Acute Care Surg. 2015; 78: 430-41. <http://dx>.doi.org/10.1097/TA.0000000000000503. | No updated guideline |
| #132 | Pathologists CAPCoA. College of American Pathologists (CAP) – Association of Directors of Anatomic and Surgical Pathology (ADASP) Interpretive Diagnostic Error Reduction in Surgical Pathology and Cytology. 2015 [cited; Available from: <http://www>.archivesofpathology.org/doi/full/10.5858/arpa.2014-0511-SA | Not published in 2015 |
| #133 | Pathologists CAPCoA. CAP-NSH Uniform Labeling of Blocks and Slides in Surgical Pathology. 2015 [cited; Available from: <http://www>.archivesofpathology.org/doi/full/10.5858/arpa.2014-0340-SA | No updated guideline |
| #134 | Pescatello LS, MacDonald HV, Ash GI, et al. Assessing the Existing Professional Exercise Recommendations for Hypertension: A Review and Recommendations for Future Research Priorities. Mayo Clin Proc. 2015; 90: 801-12. <http://dx>.doi.org/10.1016/j.mayocp.2015.04.008. | No updated guideline |
| #135 | Petrosky E, Bocchini JA, Jr., Hariri S, et al. Use of 9-valent human papillomavirus (HPV) vaccine: updated HPV vaccination recommendations of the advisory committee on immunization practices. MMWR Morb Mortal Wkly Rep. 2015; 64: 300-4. | Guideline with no systematic review |
| #136 | Physicians ACPACo. Cervical Cancer Screening in Average-Risk Women: Best Practice Advice From the Clinical Guidelines Committee of the American College of Physicians. 2015 [cited; Available from: <http://annals>.org/article.aspx?articleid=2281177 | No updated guideline |
| #137 | Physicians ACPACo. Evaluation of Patients With Suspected Acute Pulmonary Embolism: Best Practice Advice From the Clinical Guidelines Committee of the American College of Physicians. 2015 [cited; Available from: <http://annals>.org/article.aspx?articleid=2443959 | No updated guideline |
| #138 | Physicians ACPACo. Risk Assessment and Prevention of Pressure Ulcers: A Clinical Practice Guideline From the American College of Physicians. 2015 [cited; Available from: <http://annals>.org/article.aspx?articleid=2173505 | No updated guideline |
| #139 | Physicians ACPACo. Treatment of Pressure Ulcers: A Clinical Practice Guideline From the American College of Physicians. 2015 [cited; Available from: <http://annals>.org/article.aspx?articleid=2173506 | No updated guideline |
| #140 | Program in Evidence-based C. Management of early-stage Hodgkin lymphoma. 2015. | No updated guideline |
| #141 | Program in Evidence-based C. Follow-up of patients who are clinically disease-free after primary treatment for fallopian tube, primary peritoneal, and epithelial ovarian cancer. 2015. | No updated guideline |
| #142 | Program in Evidence-based C. Approach to fever assessment in ambulatory cancer patients receiving chemotherapy. 2015. | No updated guideline |
| #143 | Program in Evidence-based C. Plerixafor for autologous hematopoietic stem cell mobilization and transplantation for patients in Ontario. 2015. | No updated guideline |
| #144 | Savoia G, Coluzzi F, Di Maria C, et al. Italian Intersociety Recommendations on pain management in the emergency setting (SIAARTI, SIMEU, SIS 118, AISD, SIARED, SICUT, IRC). Minerva Anestesiol. 2015; 81: 205-25. | No updated guideline |
| #145 | Sciences IG-AioH. Guía de Práctica Clínica sobre el Manejo de la Enfermedad Meningocócica Invasiva. 2015 [cited; Available from: <http://www>.guiasalud.es/egpc/EMI/omplete/index.html | Not published in English |
| #146 | Scottish Intercollegiate Guidelines N. Diagnosis and management of epilepsy in adults. A national clinical guideline. 2003. | Duplicate |
| #147 | Scottish Intercollegiate Guidelines N. Glaucoma referral and safe discharge. A national clinical guideline. 2015. | No updated guideline |
| #148 | Shaw K, Amstutz U, Kim RB, et al. Clinical Practice Recommendations on Genetic Testing of CYP2C9 and VKORC1 Variants in Warfarin Therapy. Ther Drug Monit. 2015; 37: 428-36. http://dx.doi.org/10.1097/FTD.0000000000000192. | No updated guideline |
| #149 | Society for Vascular S. Society for Vascular Surgery practice guidelines for atherosclerotic occlusive disease of the lower extremities: management of asymptomatic disease and claudication. 2015. | No updated guideline |
| #150 | Sundararajan V, Romano PS, Quan H, et al. Capturing diagnosis-timing in ICD-coded hospital data: recommendations from the WHO ICD-11 topic advisory group on quality and safety. Int J Qual Health Care. 2015; 27: 328-33. http://dx.doi.org/10.1093/intqhc/mzv037. | No updated guideline |
| #151 | Van Poznak C, Somerfield MR, Bast RC, et al. Use of Biomarkers to Guide Decisions on Systemic Therapy for Women With Metastatic Breast Cancer: American Society of Clinical Oncology Clinical Practice Guideline. J Clin Oncol. 2015; 33: 2695-704. <http://dx.doi.org/10.1200/JCO.2015.61.1459>. | Duplicate |
| #152 | Vayssiere C, Sentilhes L, Ego A, et al. Fetal growth restriction and intra-uterine growth restriction: guidelines for clinical practice from the French College of Gynaecologists and Obstetricians. Eur J Obstet Gynecol Reprod Biol. 2015; 193: 10-8. http://dx.doi.org/10.1016/j.ejogrb.2015.06.021. | No updated guideline |
| #153 | Vilmann P, Clementsen PF, Colella S, et al. Combined endobronchial and esophageal endosonography for the diagnosis and staging of lung cancer: European Society of Gastrointestinal Endoscopy (ESGE) Guideline, in cooperation with the European Respiratory Society (ERS) and the European Society of Thoracic Surgeons (ESTS).[Erratum appears in Endoscopy. 2015 Jun;47(6):c1 Note: Vasquez-Sequeiros, Enrique [corrected to Vazquez-Sequeiros, Enrique]; PMID: 26062074]. Endoscopy. 2015; 47: 545-59. http://dx.doi.org/10.1055/s-0034-1392040 | No updated guideline |
| #154 | Wilson DP, McNeal C, Blackett P. Pediatric dyslipidemia: recommendations for clinical management. South Med J. 2015; 108: 7-14. http://dx.doi.org/10.14423/SMJ.0000000000000219. | Guideline with no systematic review |
| #155 | Wilson DP, McNeal C, Blackett P. Pediatric dyslipidemia: recommendations for clinical management.[Erratum appears in South Med J. 2016 Feb;109(2):137; PMID: 26840973]. South Med J. 2015; 108: 7-14. http://dx.doi.org/10.14423/SMJ.0000000000000219. | Guideline with no systematic review |
| #156 | Zouboulis CC, Desai N, Emtestam L, et al. European S1 guideline for the treatment of hidradenitis suppurativa/acne inversa. J Eur Acad Dermatol Venereol. 2015; 29: 619-44. http://dx.doi.org/10.1111/jdv.12966. | No updated guideline |
